# Prioritizing Work or Family? Investigating Women’s and Men’s Work-Family Decisions

**DOI:** 10.1007/s11199-026-01663-0

**Published:** 2026-07-08

**Authors:** Lianne Aarntzen, Loes Meeussen, Thekla Morgenroth, Michelle K. Ryan

**Affiliations:** 1 Social, Health and Organizational Psychology, Utrecht University, Heidelberglaan 1, Utrecht, 3584 CS The Netherlands; 2 Center for Social and Cultural Psychology, University of Leuven, Leuven, Belgium; 3 Thomas More College of Applied Sciences, Antwerp, Belgium; 4 Department of Psychological Sciences, Purdue University, West Lafayette, Indiana USA; 5 Global Institute for Women’s Leadership, The Australian National University, Australian Capital Territory, Canberra, Australia; 6 Faculty of Economics and Business, University of Groningen, Groningen, The Netherlands

**Keywords:** Gender differences, Work-family priorities, Career-family trade-offs, Cost-benefit analysis, Decision-making processes

## Abstract

Despite societal change, women and men still differ in the extent to which they prioritize work and family. Women more often reduce work hours or adjust careers to prioritize family needs, whereas men more often work overtime or decline family responsibilities to advance their careers. This study tested two possible explanations, that women and men differ in (1) how they *perceive* the costs and benefits of prioritizing work versus family; and (2) how they *weigh* these costs and benefits when deciding which domain to prioritize. We presented 625 employed participants (309 women, 316 men) with six scenarios involving work-family trade-offs. Analyses confirmed that women were more willing than men to prioritize family over work and less willing to prioritize work over family, even though both women and men were generally more willing to prioritize family. Women’s greater willingness to prioritize family was partially explained by higher perceived family benefits, and men’s greater willingness to prioritize work by lower perceived family costs. Women (compared to men) placed more weight on family benefits and less weight on career costs when considering prioritizing family over work. These findings reveal how gendered perceptions and weighting shape decision-making about work and family, which can inform policies and programs to promote more equitable career and caregiving outcomes.

**Supplementary Information:**

The online version contains supplementary material available at https://doi.org/10.1007/s11199-026-01663-0.

Despite advances in gender equality, significant disparities persist. Women continue to face structural economic disadvantages such as the gender pay gap and pension gap, leaving them less economically resilient than men (Eurostat, 2021, 2024). Women are also underrepresented in leadership positions across various industries (World Economic Forum, 2026) and in politics (Pew Research Center, 2023). Meanwhile, men are more likely to experience relational disadvantages such as weaker family bonds (Fingerman et al., 2020) and face greater difficulty in sustaining close relationships with their children after divorce (Peters & Ehrenberg, 2008). These gendered outcomes are frequently attributed to individual choices, yet an accumulating body of research suggests that women’s and men’s choices are shaped by societal norms, role expectations, and gender stereotypes rather than inherent preferences (Meeussen et al., 2021; Morgenroth et al., 2022; Ryan & Morgenroth, 2024).

One key domain in which such gendered choices manifest is the extent to which women and men prioritize their breadwinning role versus their caregiving role (Haines & Stroessner, 2019). Men are more likely than women to prioritize work at the expense of family. For example, they are more likely to work full-time (Eurostat, 2023), are less likely to interrupt their careers for a longer time when having a child (Herr et al., 2020), and are more likely to work in roles that require longer commutes or work-related travel (Wachter & Holz-Rau, 2022). Women, on the other hand, are more likely to sacrifice career opportunities for their family. For example, they more often work part-time (Eurostat, 2023), interrupt their careers (for a longer time) when they have children (Herr et al., 2020), switch to flexible or less demanding jobs (Becker & Moen, 1999), and refuse overtime for family reasons (Dahm et al., 2019). These gendered behavioral patterns, shaped by social expectations, contribute to the persistent inequalities observed in both professional and personal domains.

However, the psychological mechanisms driving these gendered work-family prioritizations remain insufficiently understood. We define work-family prioritization as deciding to give precedence to a goal in one domain over a goal in the other domain. We examine two key explanations for gender differences in willingness to prioritize career versus family: (1) women and men may differ in how they *perceive* the costs and benefits of prioritizing each domain and/or (2) women and men may differ in how they *weigh* these perceived costs and benefits when deciding what to prioritize. These mechanisms reflect how gender norms shape both what outcomes people anticipate (gendered perceptions) and the importance they assign to those outcomes in their decisions (gendered weighting).

In line with the gendered perceptions explanation, women and men may perceive the costs and benefits of career and family prioritizations differently due to societal stereotypes, vicarious experiences, and personal experiences that shape their expectations and beliefs. For instance, men may have learned through positive experiences at work that investing extra time pays off, leading them to expect greater career benefits from sacrifices for their work (e.g., higher chances of a raise or promotion). At the same time, due to pervasive cultural stereotypes portraying mothers as the superior parent (Haines & Stroessner, 2019; Liss et al., 2013; Neuenswander et al., 2023), men, compared to women, may perceive lower family costs (e.g., believing that their absence will have little impact on their children’s well-being). Thus, the perceptions explanation focuses on how gender shapes the perceived costs and benefits that prioritizing work or family will bring.

However, it is also possible that women and men perceive the costs and benefits similarly but weigh them differently due to internalized societal expectations about the prioritization of work or family (Haines & Stroessner, 2019). For example, women and men may be equally likely to believe that prioritizing their career may lead to a pay raise, but women may simply care less about this benefit. Likewise, women and men may be equally likely to believe that prioritizing their career could affect their children’s well-being, but men may consider this cost less important in their decision-making. Thus, the weighting explanation focuses on how gender shapes the weighting of costs and benefits in deciding whether to make a sacrifice for work or family. In this paper, we empirically examine the extent to which each of these two explanations jointly shape women's and men’s willingness to prioritize work or family. Below we review theoretical frameworks and empirical evidence for both mechanisms described above.

## Contextual Barriers That Shape Men’s and Women’s Work–Family Decisions

Our study examines how women and men perceive and weight the costs and benefits of work-family prioritizations. These psychological processes unfold within a broader system of gendered norms. Gender norms encompass both descriptive beliefs about what men and women typically do and prescriptive beliefs about what they should do (Burgess & Borgida, 1999; Diekman & Goodfriend, 2006; Eagly & Karau, 2002). Traditionally, men are expected to be agentic, assertive, and career-focused, whereas women are expected to be communal, nurturing, and family-oriented (Prentice & Carranza, 2002; Rudman & Fairchild, 2004). Learned early through socialization, these norms prescribe how much time and energy men and women “should” devote to work and family (Eagly & Wood, 2012) and can shape behavior even without conscious endorsement (Haines & Stroessner, 2019).

Three complementary theoretical perspectives help to situate our study within this literature. Role congruity theory (Eagly & Karau, 2002) posits that people experience penalties when they deviate from gender-typical roles, because masculine and feminine traits are perceived as zero-sum. These zero-sum perceptions shape both how others respond to people’s choices and how individuals see themselves. For example, women who work in a male sex-typed occupation are often perceived as less competent parents (Okimoto & Heilman, 2012) At the same time, people may also internalize these norms themselves, feeling guilt, doubt, or social pressure when their own choices do not align with traditional gender expectations (Aarntzen et al., 2023). Together, we expect these external reactions and internalized pressures to influence how individuals anticipate the costs and benefits of prioritizing work or family. These pressures are not limited to women; men may be especially vulnerable to backlash when violating gender norms.

Precarious manhood theory highlights that masculinity is treated as a hard-won, easily lost status that requires public validation (Vandello, et. al., 2008; Vandello & Bosson, 2013). Men are sanctioned more harshly than women for gender-atypical behaviors (Sirin et al., 2004), making them particularly sensitive to the risks of prioritizing family over work. At the same time, social norms are shifting: Fathers’ participation in childcare has increased across many countries, and flexible work arrangements and parental leave for fathers have become more readily accepted and in some contexts are encouraged (Meeussen et al., 2019; OECD, 2023). Thus, while men may still find it difficult to prioritize family, changing norms may increasingly render such choices legitimate.

The gender role prioritization model (Haines & Stroessner, 2019) further suggests that gender-atypical behavior can be accepted and even celebrated but only when individuals are still perceived as prioritizing their “primary” gendered responsibility (breadwinning for men, caregiving for women). Here, we examine how individuals themselves anticipate and weigh the costs and benefits of prioritizing family (gender-typical for women, atypical for men) or work (gender-typical for men, atypical for women).

Recent empirical work has indeed demonstrated that men perceived that prioritizing family (i.e., sacrificing work for family) involved greater costs and fewer benefits than did women (Villanueva-Moya & Expósito, 2026). Moreover, higher levels of communality in women, but not men, predicted stronger perceived benefits of prioritizing family, consistent with the notion that gender stereotype portraying women as communal caregivers shape their evaluations of prioritizing family as worthwhile (Villanueva-Moya & Expósito, 2026).

Building on these findings, we extend prior work by differentiating between work- and family-related costs and benefits rather than aggregating them into a single index. This approach allows us to capture potential nuances between career- and family-related costs and benefits, thereby enriching existing research. Moreover, previous research has not directly examined how perceptions of costs and benefits translate into willingness to prioritize work or family. Our study explicitly tests this link, addressing the possibility that women and men differ not only in how they perceive the costs and benefits of work–family prioritizations but also in how strongly these perceptions guide their willingness to prioritize work or family.

With our approach to distinguish between career- and family-related costs and benefits, we connect with the work-family interface literature, which has traditionally emphasized that the time and energy spent on one role can come at the expense of another (Greenhaus & Powell, 2006; Van Steenbergen et al., 2007). This conflict can manifest not only as reduced time but also as depleted energy, impaired performance, and psychological strain across roles. For example, feeling emotionally drained from work may lead to fewer contributions at home. As such prioritizing of work or family does not solely generate within-domain benefits (e.g., prioritizing work resulting in a promotion) but also cross-domain costs (e.g., prioritizing work resulting in a higher care burden for the partner).

At the same time, work-family enrichment research demonstrates that prioritization of one domain can also produce cross-domain benefits through increased energy, skills, time efficiencies, or psychological resources (Greenhaus & Powell, 2006; Van Steenbergen et al., 2007). For example, coming home from work feeling energized enables one to participate more actively in family activities. Therefore, rather than treating prioritizing work or family as a strictly zero-sum trade-off we examine costs and benefits both within-domain (e.g., prioritizing work as beneficial for work) and cross-domain (e.g., prioritizing work as beneficial for family).

## Explaining Gender Differences in Work/Family Prioritizations

In line with various decision-making theories, we argue that people tend to make decisions based on both their expectations about likely outcomes and the value they attach to such outcomes. This principle is foundational to several theoretical frameworks on decisions, including expectancy-value theory (Wigfield & Eccles, 2000) and the theory of planned behavior (Ajzen, 1991). Both suggest that behavior is guided by a combination of perceived outcomes, and by how much individuals care about these potential outcomes. In other words, when considering a sacrifice individuals ask: “Will this pay off or be harmful?” (i.e., will it result in benefits or costs?) and “Does it matter to me?” (i.e., how valuable and costly are these benefits and costs?).

Expectancy-value theory, for instance, posits that individuals are more likely to pursue a goal when they believe they can succeed (expectancy) and when they value the potential outcomes (value). This expectancy-value mechanismhas been shown in the domain of educational and career choices. For example, girls are more likely to pursue STEM fields when they believe they can do well and find the outcomes of STEM careers (e.g., intellectual stimulation, societal impact) personally meaningful (Eccles & Wang, 2016). Similarly, the theory of planned behavior argues that people’s intentions are shaped by their attitudes toward the behavior (e.g., is this behavior desirable or important?) and their perceived behavioral control (e.g., are the outcomes feasible and/or worth the cost?). This theory has been applied to understand a range of life decisions. For example, people’s intentions to engage in various leisure activities—such as jogging or mountain climbing—were accurately predicted by their attitudes, subjective norms, and perceived behavioral control (Ajzen & Driver, 1992), supporting the idea that people weigh both the value and feasibility of a behavior when making decisions. Although expectancy–value theory and the theory of planned behavior have been widely used to explain educational, health, and career choices, they have rarely been applied to understand gendered work–family decisions, leaving an important gap in understanding the psychological mechanisms underlying gender differences in these choices. By explicitly extending these decision-making frameworks to men’s and women’s anticipated costs, benefits, and value priorities, our study provides a novel test of how these mechanisms shape gender differences in work–family prioritizations.

Building on these foundations, our study distinguishes between two psychological processes that may explain gender differences in willingness to prioritize work or family: *perceptions* and *weighting*. Perceptions reflect how women and men differ in their expectations of the costs and benefits that prioritization behaviors bring, similar to constructs such as perceived behavioral control and expectancies. For example, individuals may differ in whether they expect that working long hours will lead to a meaningful pay raise, or whether taking parental leave will negatively affect their career progressions (Morgenroth et al., 2022).

Weighting, on the other hand, reflects how women and men differ in how much importance they assign to these costs and benefits, comparable to concepts such as value and attitudes. For instance, even if both believe that working overtime increases the chance of a pay raise, they may differ in how much they weigh this raise in their decision to sacrifice. By exploring these two mechanisms, our study contributes to a better understanding of how gendered perceptions and weighting of costs and benefits shape men’s and women’s everyday choices, thereby helping to explain persistent gender inequalities.

### Gendered Perceptions of Costs and Benefits

The extent to which individuals expect to gain benefits or incur costs from their work-family prioritizations is shaped by various factors, including previous experiences when engaging in similar behaviors, observations of the costs and benefits faced by same-gender reference groups, and gender stereotypes about parental and worker competencies. First, empirical evidence indicates that the time and energy that women invest in work tend to yield lower rewards than those of men (Ellemers, 2014). Women receive fewer financial rewards, such as lower salaries (World Economic Forum, 2025) and fewer bonuses (Kulich et al., 2011). Moreover, they experience fewer psychological rewards compared to men, including getting less credit in teamwork (Heilman & Haynes, 2005), lower levels of co-worker support (Faniko et al., 2017), fewer representation opportunities (Johnson et al., 2017), and lower competence ratings (Moss-Racusin et al., 2012). These disparities likely shape women’s expectations of success—or lack thereof—when considering prioritizing their career over their family. Indeed, correlational research indicates that in male-dominated fields, women are less willing than men to make sacrifices for their career due to their lower expectations that these sacrifices will pay off, as they often experience workplace gender discrimination and a lower perceived fit with people higher-up the ladder (Meeussen et al., 2021). Thus, we expect women to perceive fewer career benefits when deciding to make a sacrifice for work.

Second, gender stereotypes and backlash may also shape how fathers and mothers anticipate the family-related costs and benefits of work-family prioritizations. When considering giving priority to work over family—such as working overtime to perform good at work—mothers may perceive higher family-related costs than fathers, given societal norms that position them as the primary and most essential caregivers (Liss et al., 2013; Morgenroth & Heilman, 2017; Neuenswander et al., 2023; Park et al., 2010). Conversely, when considering giving priority to family over work—such as reducing work hours to support family—women may perceive greater benefits for their family than men. Prevailing beliefs that mothers provide superior care make it easier for fathers to prioritize work while mothers may feel they are depriving their children of their presence. Moreover, mothers who deviate from traditional caregiving expectations by prioritizing their careers often face social backlash. For example, women who do not take maternity leave are perceived more negatively in family-related contexts than mothers who take maternity leave (Morgenroth & Heilman, 2017). Based on this previous research, we predict that, on average, men will appraise prioritizing their work as bringing lower costs to their family than women while they also appraise prioritizing family as bringing fewer benefits to their family, making men more willing to sacrifice for work and less willing to sacrifice for their family than women.

Third, the literature is mixed in predicting whether women or men experience higher career costs when prioritizing family. On the one hand, studies show that men who do not uphold the masculine “ideal worker” image are not only seen as poor workers but also are less respected (Heilman & Wallen, 2010; Rudman & Mescher, 2013). On the other hand, studies also show that women are judged equally harshly at work and their parental status can lead to being held to stricter standards at work than fathers (Fuegen et al., 2004). This stricter scrutiny likely intensifies when women make sacrifices for their families, because these sacrifices would further highlight their parental role. Therefore, we will *explore* the extent to which gender impacts the perceived career costs of making sacrifices for one’s family (without a directional hypothesis).

Finally, men may be more inclined than women to perceive family benefits from investing in their career. Unlike women, who often experience a double bind—balancing both career and caregiving expectations—men’s career investments may be more seamlessly aligned with their socially expected breadwinner role (Haines & Stroessner, 2019; Okimoto & Heilman, 2012). As a result, men may see sacrifices for their work not only as professionally beneficial but also as serving their family’s (financial) well-being. As such, men (more than women) may perceive greater family benefits when they sacrifice for work.

To summarize, we expect that men will perceive more career benefits, fewer family costs and more family benefits than women when considering a sacrifice for work. Furthermore, we expect women to perceive more family benefits than men when considering a sacrifice for family. We will also explore potential gender differences in perceived career costs. These gendered perceptions are expected to explain (i.e., mediate) differences in men’s and women’s willingness to make sacrifices.

## Gendered Weighting of Costs and Benefits

Women and men often navigate work-family decisions within the constraints of societal expectations and internalized values. These expectations and internalized roles may shape how willing individuals are to prioritize their family or their work. Empirical research supports this notion, demonstrating that women and men tend to prioritize different life domains. Studies consistently find that men, on average, place greater emphasis on career success and financial provision, whereas women prioritize caregiving and family well-being (Block et al., 2018; Hakim, 2006; Konrad & Mangel, 2000). For example, research on work values shows that men are more likely to endorse extrinsic rewards (e.g., salary, promotions), while women place greater value on work-family balance (Hakim, 2006). These findings suggest that when deciding whether to prioritize career or family, women and men may weight costs and benefits differently, reflecting different role internalizations and value orientations.

This divergence in prioritization is also visible in how fathers and mothers respond to work-family conflict. When both fathers and mothers experience similar levels of conflict, mothers are more likely to reduce work hours or seek family-friendly jobs (Young & Schieman, 2018). Such patterns imply that family obligations may carry greater subjective weight for women than for men.

Building on this reasoning, we propose that women and men differ not only in how they perceive the costs and benefits of work-family prioritizations, but also in how heavily they weight these considerations when making decisions. Specifically, we expect women to assign greater weight to family-related outcomes: They will place more importance on family benefits when considering prioritizing their family over their work, and more importance on family costs when considering prioritizing their work over their family. As a result, women may be more willing to make prioritize family and less willing to prioritize work compared to men. Conversely, we expect men to assign greater weight to career-related outcomes: they will give more weight to career benefits when considering a sacrifice for work, and to career costs when considering prioritizing family, relative to women.

### The Current Study

This is the first study, to our knowledge, to empirically investigate whether a gendered perceptions and weighing of work-family benefits and costs underlies women's and men’s willingness to prioritize work or family. Specifically, we measure perceived costs and benefits as the central constructs and test two possible complementary processes—mediation and moderation—through which they may shape gendered willingness to prioritize work or family. First, drawing on the *gendered perceptions* explanation, we predict that women and men differ in how high they perceive the costs and benefits of prioritizing work or family. This suggests a mediation model, in which gender differences in willingness to prioritize work or family are explained by differences in perceived benefits and costs. Second, based on the *gendered weight* explanation, we propose that even when women and men perceive similar costs and benefits, they differ in how strongly these perceptions influence their willingness to prioritize work or family. This suggests a moderation model, in which gender moderates the relationship between perceived costs and benefits and willingness to prioritize work or family. Both explanations may hold true simultaneously and explain the gendered willingness to prioritize work or family.

We present participants with different prioritization behaviors for work or family and examine how they perceive and weigh the costs and benefits when deciding their willingness to prioritize their work or their family. Specifically, we hypothesize the following:

#### H1a

Men will be more willing than women to prioritize work over family.

#### H1b

Women will be more willing than men to prioritize family over work.

### Mediation Hypotheses

#### H2a

The gender difference in willingness to prioritize family will be mediated by perceived family benefits, with women perceiving higher family benefits than men.

#### H2b

The gender difference in willingness to prioritize work will be mediated by perceived career benefits, with men perceiving higher career benefits than women.

#### H2c

The gender difference in willingness to prioritize work will be mediated by perceived family costs, with men perceiving lower family costs than women.

#### H2d

The gender difference in willingness to prioritize work will be mediated by perceived family benefits, with men perceiving higher family benefits than women.

#### **H2e** (*Exploratory*)

We will explore whether gender differences in willingness to prioritize family are mediated by perceived career costs, given mixed evidence about whether such costs are perceived higher by men or by women.

### Moderation Hypotheses

#### H3a

The effect of perceived family costs on willingness to prioritize work will be stronger for women than for men.

#### H3b

The effect of perceived career benefits on willingness to prioritize work will be stronger for men than for women.

#### H3c

The effect of perceived family benefits on willingness to prioritize family will be stronger for women than for men.

#### H3d

The effect of perceived career costs on willingness to prioritize family will be weaker for women than for men.

## Method

### Participants

Using a Prolific sample, 633 heterosexual working women and men were invited to participate in the survey. All participants were fluent in English. Seven participants were excluded from the analyses because they missed more than one of three attention checks, and one participant was excluded because they identified as genderqueer while this study focused on gender differences between participants who identified as women or men.

The final sample consisted of 625 participants (309 women, 316 men) between the ages of 18 and 53 (*M* = 34.86, *SD* = 5.38). Most participants had children who were living at home (*n* = 586; 93.76%), with between one and six children (*M* = 1.78, *SD* = 0.86), and the age of their youngest child ranged from 0 to 25 (*M* = 5.31, *SD* = 4.65). Most participants (*n* = 549; 87.9%) were currently in a relationship and 65.8% (*n* = 411) of participants were married. On average, participants worked 37.75 hours per week (*SD* = 10.75, range 0–75), and they were on average 14.76 years (*SD* = 6.74) on the labor market. They spent on average 29.34 hours per week on household and care responsibilities (*SD* = 24.27). Participants without children reported significantly fewer hours (*M* = 13.23, *SD* = 9.90) than participants with children (*M* = 30.42, *SD* = 24.57), *t*(75.33) = 9.12, *p* < .001. Most participants were U.K. (*n* = 212; 33.9%) or U.S. (*n* = 238; 38.1%) nationals.

An a priori power analysis in G*power indicated that 612 participants were needed to detect a medium effect (*f* = 0.25) at 95% power when analyzing this study as a gender (between-participants) × prioritization domain (between-participants) design. However, after finishing data collection and consulting with a statistical expert, we determined that power could be significantly improved by applying a multilevel modeling approach, nesting the participant’s rating on the different sacrificing behaviors within participants. Another advantage of this approach is that it retains the richness of these different behaviors (e.g., relocating for a job versus occasionally working outside regular hours) rather than forcing them into a single aggregated measure and introducing measurement error. Moreover, participants' perceptions of the prioritizations (i.e., perceived career costs, family costs, career benefits, family benefits) varied substantially across scenarios, making a single reliability estimate problematic. Thus, a multilevel approach enhanced both statistical rigor and theoretical precision.

### Procedure and Measures

Participants evaluated six different work-family sacrificing behaviors for their perceived costs and benefits for both their career and their family, and their willingness to engage in these behaviors. To ensure that the behaviors included were relevant across various jobs and countries, we first conducted a pilot study. Here, 80 employed women and men (48.8% women) listed three instances in which they prioritized work over family that benefited their career at a potential cost to their family and three instances in which they prioritized family over work that benefited their family at a potential cost to their career. We selected the most frequently mentioned and widely applicable sacrificing behaviors for the main study (see Table S1 in the online supplement for details). Importantly, these behaviors closely mirror those used in Dahm et al. (2019), who developed a validated work–life trade-off scale that captures both minor and major trade-offs (showing minor trade-offs to sometimes be even more important for outcomes such as life satisfaction than major trade-offs). Consistent with their findings, our set included both types of behaviors. Three behaviors reflected prioritizing work behaviors—occasionally working outside regular hours (minor), accepting additional responsibilities at work (major), and relocating for a job (major)—while the other three reflected prioritizing family behaviors— missing work to care for a sick family member (minor), reducing work hours for family reasons (major), and choosing not to pursue career-enhancing opportunities (major). In the main study, participants assessed six behaviors presented in randomized order.

For each behavior, participants rated the perceived benefits for their career and perceived benefits for their family on two separate 7-point scales (1 = *not at all beneficial*, 7 = *very beneficial*, e.g., “If you occasionally worked outside regular working hours [e.g., long hours, evenings, weekends], to what extent would this be beneficial for your career?”). They also rated the perceived costs for their career and perceived costs for their family on two additional 7-point scales (1 = *not at all costly*, 7 = *very costly*; e.g., “If you occasionally worked outside regular working hours [e.g., long hours, evenings, weekends], to what extent would this be beneficial for your family?”). The same measures of perceived costs and benefits were entered as mediators (testing their underlying role in shaping willingness) in the mediation models and as moderators (testing how much weight is attributed to them in shaping willingness) in the moderation models to reflect their distinct theoretical role. Finally, they indicated their willingness to engage in the behavior on a 7-point scale ranging from 1 (*not at all*) to 7 (*very much*). After completing these ratings, participants provided demographic information. Some additional exploratory measures were included but not analyzed because they were not the focus of the current study (e.g., anticipated guilt; a full overview of the survey can be asked from the first author. All participants provided informed consent before starting the study. Furthermore, this study received ethical clearance from the Psychology Ethics Committee at the University of Exeter (ID: eCLESPsy001203 v3.0) where the third and fourth authors were affiliated at the time.

### Analytical Approach

All hypotheses were analyzed as linear-mixed models with the six behaviors nested within participants. These models were assessed using the lavaan package in R (Rosseel, 2012), incorporating random intercepts. We chose not to include random slopes in models with three-way interactions due to increased model complexity. Note that significance and effect direction remained stable in models where random slopes were feasible. All models were estimated using full information maximum likelihood estimation (FIML). For all cross-level interactions, a stepwise approach was applied: (1) examining the intercept-only model, (2) adding a random intercept and fixed slope model including the main effects, and (3) adding the cross-level interaction. Results of this stepwise approach are detailed in the online supplement (Tables S2-S6). If interactions were significant, pairwise comparisons with Bonferroni corrections for multiple comparisons were conducted.

H1 was tested by performing cross-level interaction analyses between gender and prioritization domain on willingness to engage in the prioritization behavior. H2 was tested in two steps. First, the main effects of gender on perceived family benefits, perceived family costs, perceived career benefits, and perceived career costs were examined separately for prioritizations made for family and career. Second, two multilevel mediation models (2-1-1 mediation) were estimated to test the indirect effects of gender on willingness to prioritize work or family via perceived costs and benefits. One mediation model examined the effects for prioritizing work, while the other focused on prioritizing family. To test H3a-e, four two-way cross-level interaction analyses were entered simultaneously to examine the moderating role of perceived costs and benefits in the relationship between gender and willingness to engage in the prioritization behavior. Separate models were estimated for willingness to prioritize career and willingness to prioritize family (the included two-way interactions in both models were: gender × perceived family benefits, gender × perceived family costs, gender × perceived career benefits, and gender × perceived career costs).

All primary analyses discussed below were conducted on our full sample. Most participants had children living in their household; only 6.24% did not. Additional analyses restricted to participants with children in the household produced similar effect directions and significance levels. All analyses were conducted both with and without controlling for background variables. Results are reported without these covariates, however, including number of children, household type (single- or dual-adult), and participants’ age as covariates did not alter the direction or significance of effects.

## Results

### Descriptive Statistics

Table 1 presents means, standard deviations, and correlations between the measures for women and men. *T*-value difference tests suggest that women and men do not differ in perceived benefits or costs of prioritizations (when not distinguishing domains) or in willingness to prioritize in general (regardless of the domain).

**Table 1:** Descriptives of and Correlations Between Measures for Women and Separately

Variable	*M* (*SD*) _*women*_	*M* (*SD*) _*men*_	*t*(*df*) _gender difference_	*p-*value	1	2	3	4	5
1. Perceived career benefits	3.97 (0.63)	4.02 (0.69)	0.97 (623)	.331	–	.16*	.14	.56***	.39***
2. Perceived career costs	3.75 (0.85)	3.66 (0.81)	-1.28 (623)	.201	.20***	–	.43***	.19*	.07
3. Perceived family costs	4.19 (0.85)	4.07 (0.81)	-1.92 (623)	.055	.09	.39***	–	.01	.05
4. Perceived family benefits	4.30 (0.85)	4.19 (0.81)	-1.81 (623)	.071	.46***	.29***	.07	–	.47***
5. Willingness to prioritize	4.52 (0.85)	4.49 (0.81)	-0.65 (623)	.515	.36***	.04	− .02	.48***	–

*Note*. Correlations below the diagonal are for women, correlations above the diagonal are for men

### Are Men More Willing to Prioritize Their Work and Women More Willing to Prioritize Their Family?

Our analyses showed a main effect of prioritization domain, *B* = 0.57, *SE* = 0.06, *t*(3750) = 9.63, *p* < .001, *η²ₚ* = .02, but not of gender, *t*(3750) = 0.63, *p* = .527. on people’s willingness to engage in prioritization behaviors. Participants were generally more willing to prioritize their family than their career (*ΔM* = -0.57, *SE* = 0.05, *z =* -9.63, *p <* .001). The absence of a main effect of gender indicates that there is no overall difference in how willing women and men are to engage in prioritization behaviors in general (regardless of the domain).

**Fig. 1:**
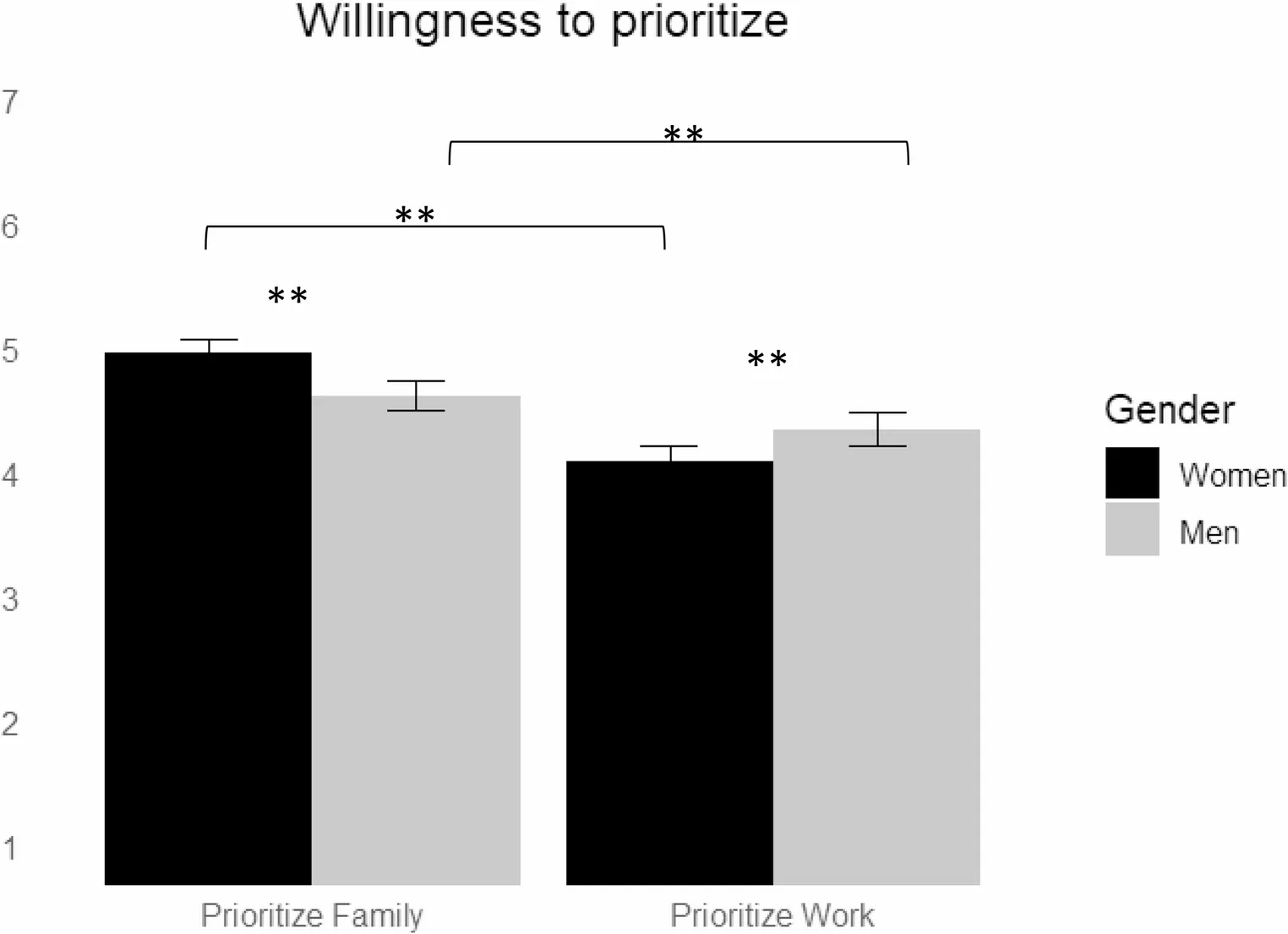
Willingness to prioritize by gender and prioritization domain. Error bars represent 95% confidence intervals. **p < .01

Furthermore, analyses revealed a significant gender × prioritization domain interaction, *B* = 0.60, *SE* = 0.12, *t*(3750) = 5.13, *p* < .001, *η²ₚ* = .01. Supporting H1, men were more willing than women to prioritize their work (*ΔM* = 0.27, *SE* = 0.08, *z* = 3.18, *p* = .001). In contrast, women were more willing than men to prioritize their family (*ΔM* = -0.34, *SE* = 0.08, *z* = -4.08, *p* < .001). Finally, both women and men were more willing to prioritize their family than to prioritize their career (Men: *ΔM* = -0.27, *SE* = 0.08, *z* = -3.26, *p* = .001; Women: *ΔM* = -0.88, *SE* = 0.08, *z* = -10.44, *p* < .001). Means and confidence intervals per prioritization domain and gender are displayed in Fig. 1.

### Do Women and Men Differ in the Costs and Benefits They Perceive in Prioritizing for Work or Family (Gendered Perceptions Explanation)?

**Fig. 2:**
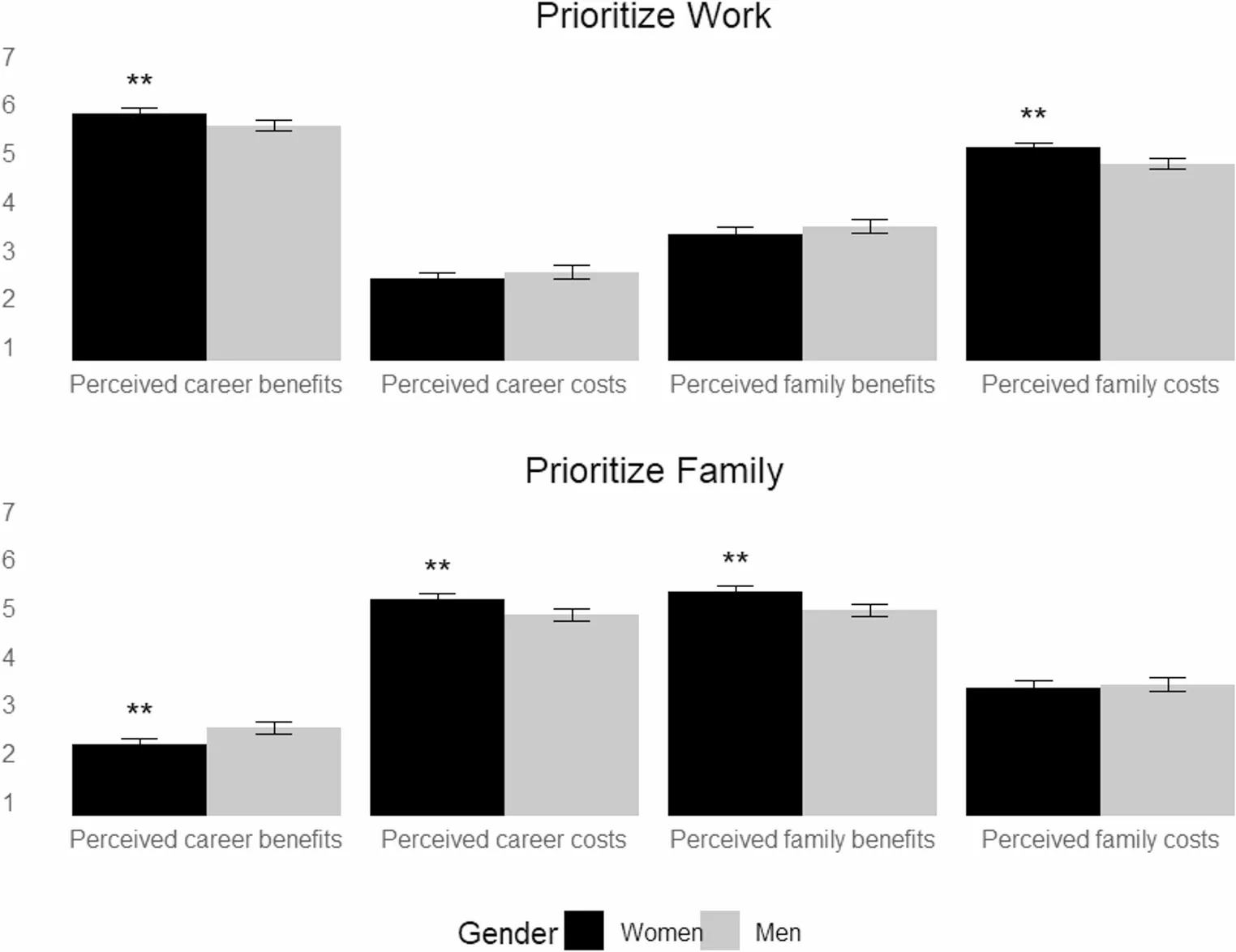
Perceived family/career benefits and costs by gender and prioritization domain. Error bars represent 95% confidence intervals. **p < .01

A first explanation for this gendered willingness to prioritize is that women and men differ in the costs and benefits they perceive from a prioritizing their work or family. Results testing these gendered costs/benefitsare summarized in Fig. 2. First, regarding prioritizing the work domain, gender significantly predicted both perceived career benefits, *B* = 0.23, *SE* = 0.08, *t*(625) = 2.90, *p* = .004, *η²ₚ* = .01, and perceived family costs, *B* = 0.33, *SE* = 0.08, *t*(625) = 3.93, *p* < .001, *η²ₚ* = .02. Specifically, in line with H2c, women perceived prioritizing their work as bringing more costs for their family than men. However, contrary to H2b, women also perceived such prioritizations as bringing more career benefits than men. No significant effects were found for perceived career costs, *t*(625) = -1.45, *p* = .147, or perceived family benefits, *t*(625) = -1.64, *p* = .102.

Second, regarding prioritizing the family domain, gender was found to significantly predict perceived career benefits, *B* = -0.33, *SE* = 0.09, *t*(625) = -3.87, *p* < .001, *η²ₚ* = .02, perceived career costs, *B* = 0.31, *SE* = 0.08, *t*(625) = 3.49, *p* < .001, *η²ₚ* = .02, and perceived family benefits, *B* = 0.38, *SE* = 0.09, *t*(188) = 4.28, *p* < .001, *η²ₚ* = .01, but not perceived family costs, *t*(625) = -0.68, *p* = .501. Supporting H2a, women perceived prioritizing their family as yielding greater benefits to their family than men. Addressing our exploratory question (H2e) on how gender impacts the perceived career costs of prioritizing family behaviors—where existing literature is mixed—we found that women viewed these family prioritizations as more detrimental to their careers than men, perceiving them as less beneficial and more costly to their career. Note that all effects remain identical (both significance level and direction of effects) when conducting a 2 (sacrifice domain) × 2 (gender) interaction analysis and examining pairwise comparisons with Bonferroni corrections.

### Can Gendered Perceptions Explain Gendered Work/Family Prioritizations?

**Fig. 3:**
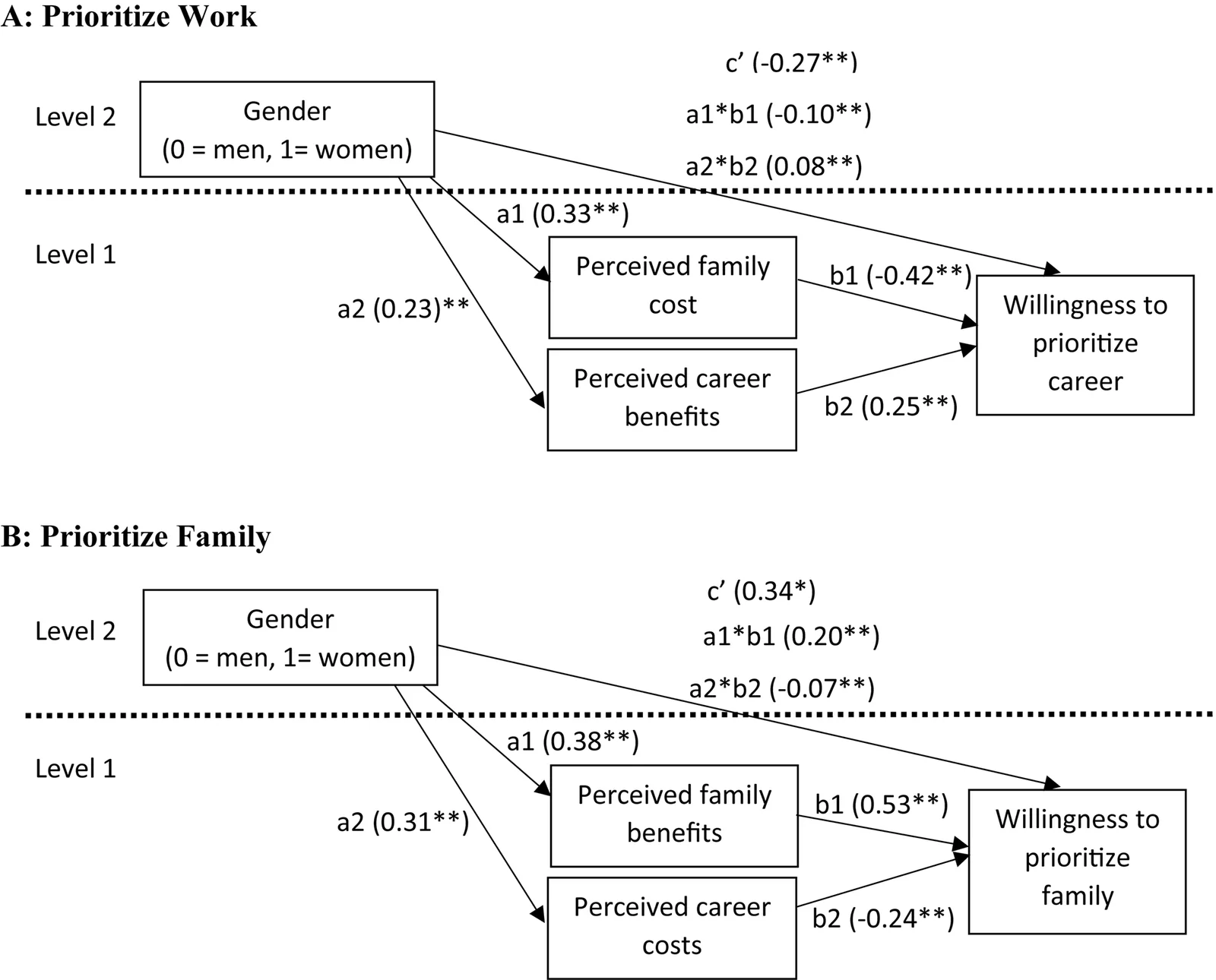
Mediation of the effects of the cost-benefit analyses on the relationship between gender and willingness to prioritize work or family. Panel A demonstrates significant indirect effects of perceived family costs and career benefits on the relationship between gender and willingness to prioritize work. Panel B demonstrates significant indirect effects of perceived family benefits and career costs on the relationship between gender and willingness to prioritize family. *p < .05; **p <.01

The findings discussed in the previous paragraph confirm that women and men perceive the costs and benefits of prioritizing for work or family differently. The question remains whether such gendered cost/benefit perceptions underlie gender differences in willingness to engage in a prioritization. Therefore, the two hypothesized mediation models were tested. In these models, perceived costs and benefits act as mediators (i.e., “perceptions”), whereas in the later section on gendered weights we examine them as moderators (“weights”). The first model examined whether women’s higher perceived family costs and unexpectedly also their higher (instead of lower) perceived career benefits explain their lower willingness to prioritize their work compared to men. We excluded the hypothesized perceived family benefits from this model as the previous section showed that gender did not significantly predict this perception. The second model examined whether women’s higher perceived family benefits and lower perceived career costs explain their higher willingness to sacrifice for family compared to men. Note that both models included the hypothesized costs and benefits simultaneously to estimate their unique and potentially opposing effects on willingness to sacrifice. A full overview of the results of both models is presented in Fig. 3. Note that both models were fully saturated, meaning that all possible paths were estimated, leaving zero degrees of freedom. As a result, fit indices could not be reliably assessed. We deliberately chose not to remove covariances or correlations to artificially increase degrees of freedom, as our primary focus was on testing the theoretical models rather than optimizing predictive accuracy.

The first model examined willingness to prioritize work (see Fig. 3, Panel A). Supporting H3a, there was a significant indirect effect of gender on willingness to prioritize work through perceived family costs (*B* = -0.10, *SE* = 0.03, *p* = .004), showing that women perceived higher family costs than men (*B* = 0.33, *SE* = 0.08, *p* < .001) and therefore were less willing to prioritize their work (*B* = -0.42, *SE* = 0.03, *p* < .001). Contrary to H3b, there was a significant indirect effect of gender on willingness to prioritize work through perceived career benefits (*B* = 0.08, *SE* = 0.02, *p* = .001), showing that women perceived higher career benefits than men (*B* = 0.23, *SE* = 0.08, *p* = .004) and this related to higher willingness prioritize their work (*B* = 0.25, *SE* = 0.03, *p* < .001). The indirect effect of perceived family costs on willingness to prioritize was stronger than of perceived career benefits, partially explaining why women were still less willing to prioritize their work than men. Additionally, a direct effect of gender on willingness prioritize work remained significant (*B* = -0.27, *SE* = 0.09, *p* = .004), suggesting that gender differences in willingness to prioritize persist beyond perceptions of family costs and career benefits.

The second model examined willingness to prioritize family (see Fig. 3, Panel B). Supporting H3c, there was a significant indirect effect of gender on willingness to prioritize family through perceived family benefits (*B* = 0.20, *SE* = 0.05, *p* < .001), showing that women perceived higher family benefits than men (*B* = 0.38, *SE* = 0.085, *p* < .001) and were therefore more willing to prioritize their family (*B* = 0.53, *SE* = 0.02, *p* < .001). Additionally, there was a significant indirect effect of gender on willingness to prioritize for family through perceived career costs (*B* = -0.07, *SE* = 0.02, *p* = .001), showing that women perceived higher career costs than men (*B* = 0.31, *SE* = 0.09, *p* = .001) and were therefore less willing to prioritize their family (*B* = -0.23, *SE* = 0.03, *p* < .001). The indirect effect of perceived family benefits on willingness to prioritize family was stronger than the effect of perceived career costs, partially explaining why women were still more willing to prioritize their family than men. Finally, a direct effect of gender on willingness to prioritize family remained significant (*B* = 0.34, *SE* = 0.08, *p* < .001), suggesting that gender differences in willingness to prioritize for family persist beyond perceptions of family benefits and career costs.

Together, these results show that men are more willing to prioritize their career due to lower perceived family costs, although they also see lower career benefits thereof than women. Women are more willing to prioritize family due to higher perceived family benefits, although they also see higher career costs thereof than men. However, these gendered perceptions do not fully explain the gender differences in willingness to prioritize work and family, suggesting other factors at play. Therefore, we next turn to the *gendered weights explanation*, to examine to what extent men and women weigh costs and benefits differently in their decision to prioritize work or family.

### Can Gendered Weighting Explain Gendered Work/Family Prioritizations?

An additional possible explanation for this gendered willingness to prioritize family or work is that women and men differentially weight the perceived costs and benefits in their willingness to sacrifice. Here we test the same perceived costs and benefits but now moderating the effect of gender on willingness to prioritize work or family, thereby making the “weighting” process empirically visible. In a first mixed model, we tested four cross-level interactions between gender and perceived costs and benefits on willingness to prioritize work (gender × career costs, gender × family costs, gender × career benefits, gender × career costs). None of the two-way interactions reached significance (see Table 2).

**Table 2:** Linear Mixed Model Predicting Willingness to Prioritize Work

Predictors	*B*-weight	*SE*	*df*	*t*-value	*p*-value	partial *η²*
Intercept	3.12	0.28	1719.17	11.13	< .001	
Main effects						
Gender	0.33	0.41	1711.75	0.80	.422	.00
Career benefit	0.27	0.04	1810.33	6.58	< .001	.02
Career cost	-0.06	0.04	1729.47	-1.63	.097	.00
Family cost	-0.24	0.03	1855.78	-7.47	< .001	.03
Family benefit	0.31	0.03	1824.72	9.28	< .001	.05
Two-way interactions						
Gender × Career benefit	-0.05	0.06	1806.01	-0.85	.397	.00
Gender × Career cost	0.02	0.05	1716.67	0.51	.614	.00
Gender × Family cost	-0.05	0.05	1864.41	-1.13	.259	.00
Gender × Family benefit	-0.02	0.05	1822.67	-0.41	.680	.00

*Note*. Gender is coded as 0 = Men, 1 = Women. Career benefit / Career Cost / Family cost and Family benefit all indicate participant perceptions

In a second mixed model, we tested four cross-level interactions between gender and the perceived costs and benefits on willingness to prioritize family (gender × career costs, gender × family costs, gender × career benefits, gender × career costs). Two of the four interactions reached significance (see Table 3); the interaction between gender and career costs and the interaction between gender and family benefits on willingness to prioritize family.

**Table 3:** Linear Mixed Model Predicting Willingness to Prioritize Family

Predictors	*B*-weight	*SE*	*df*	*t*-value	*p*-value	partial *η²*
Intercept	3.99	0.26	1793.00	15.49	< .001	
Main effects						
Gender	-0.81	0.37	1803.00	-2.17	.030	.00
Career benefit	-0.00	0.04	1803.00	-0.05	.958	.00
Career cost	-0.25	0.03	1864.00	-7.87	< .001	.03
Family cost	-0.07	0.03	1823.00	-2.37	.018	.00
Family benefit	0.43	0.03	1840.00	14.81	< .001	.11
Two-way interactions						
Gender × Career benefit	0.04	0.05	1832.00	0.77	.444	.00
Gender × Career cost	0.13	0.04	1856.00	2.94	.003	.01
Gender × Family cost	-0.06	0.04	1830.00	-1.40	.161	.00
Gender × Family benefit	0.10	0.04	1820.00	2.38	.018	.00

*Note*. Gender is coded as 0 = Men, 1 = Women. Career benefit / Career Cost / Family cost and Family benefit all indicate participant perceptions

**Fig. 4:**
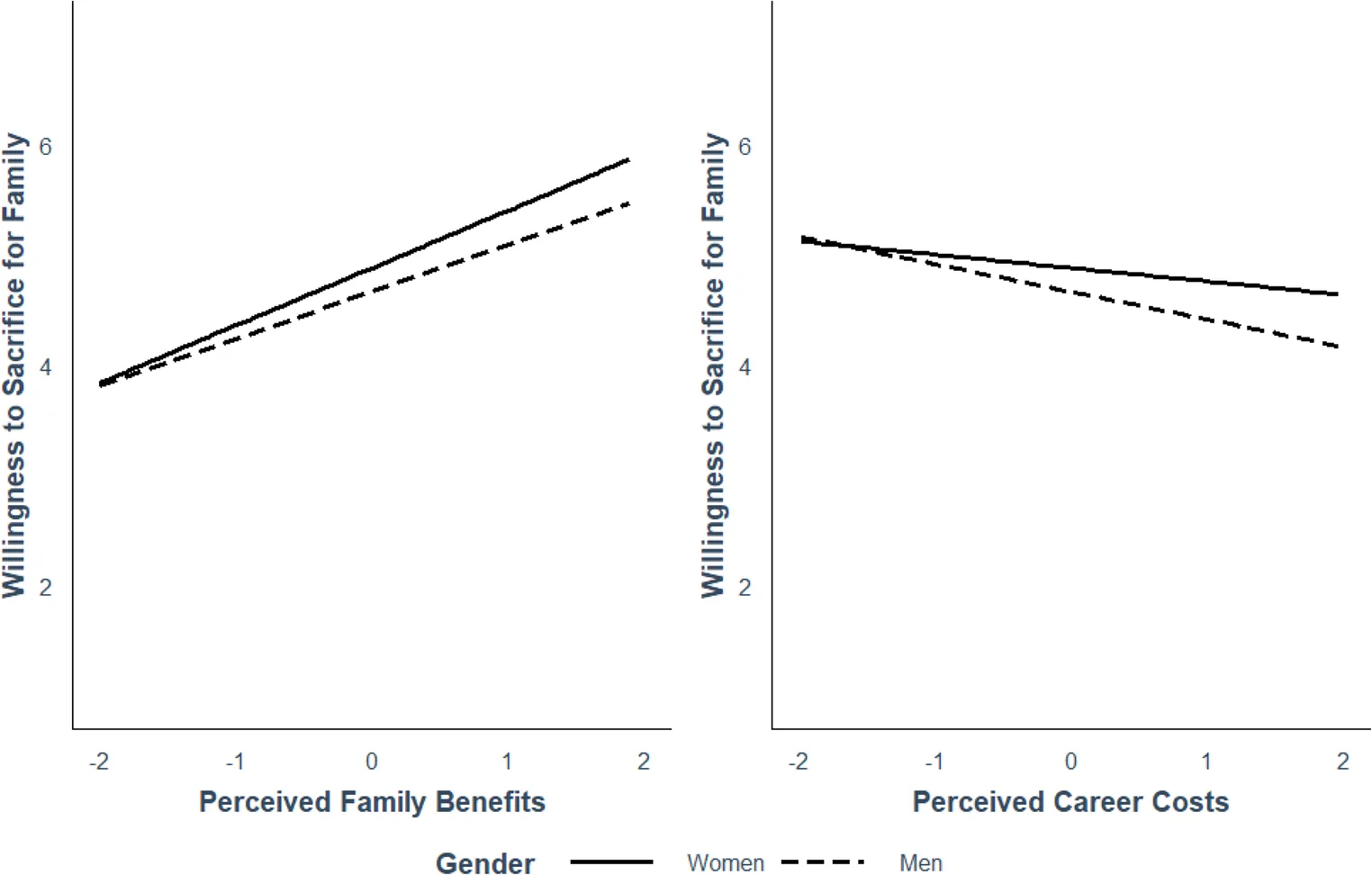
Mixed model two-way interactions between perceived family benefits and gender on willingness to prioritize family (left panel) and perceived career costs and gender on willingness to prioritize family (right panel). The predictors Perceived Family Benefits and Perceived Career Costs are mean-centered. The standard deviation for perceived family benefits is 1.93, and for perceived career costs is 1.74

To interpret these significant two-way interactions, simple slopes with mean-centered predictors were analyzed using the “interaction” package in R (Long, 2024). Figure 4 illustrates the pattern of both two-way interactions. Simple slopes revealed that when perceived family benefits are low, there is no significant difference in women's and men’s willingness to prioritize family (-1 *SD*; *B* = 0.03, *SE* = 0.11, *p* = .79). However, when perceived family benefits are high, women are more willing to prioritize family than men (+ 1 *SD*; *B* = 0.40, *SE* = 0.11, *p* < .001. Note that for both women and men, higher perception of career benefits resulted in higher willingness to prioritize family but for women this slope was steeper (*Women*; *B* = 0.52, *SE* = 0.03, *p* < .001; *Men*; *B* = 0.42, *SE* = 0.03, *p* < .001).

In line with Hypothesis 3d, this suggests that women weigh the benefits for their family more in their willingness to prioritize than men. Similarly, simple slopes revealed that when perceived career costs are low, there is no significant difference in women’s and men’s willingness to prioritize family (-1 *SD*; *B* = -0.01, *SE* = 0.11, *p* = .94. However, when perceived career costs are high, men are less willing to prioritize family than women (+ 1 *SD*; *B* = 0.44, *SE* = 0.11, *p* < .001). Note that for both women and men higher perception of career costs resulted in lower willingness to prioritize family but for men this slope was steeper (*Women*; *B* = -0.12, *SE* = 0.03, *p* < .001; *Men*; *B* = -0.25, *SE* = 0.03, *p* < .001).

In line with Hypothesis 3e, this suggests that men weight the costs for their career more in their willingness to prioritize family than women. Together, these results suggest that both women and men consider perceived family benefits and career costs when considering engaging in a prioritization of their family over their work, but the relative weight they give to these outcomes differs: women are more sensitive to family benefits, whereas men are more sensitive to career costs.

### Gender Differences in Perceived Costs and Benefits and Willingness to Prioritize

To ensure that the observed gender patterns were not driven by extreme sacrifices—either very small (e.g., missing work for a sick family member) or very large (e.g., moving one’s family for a job)—we examined means and standard deviations separately for each of the six situations (for an overview of results, see Table 4), alongside the aggregated multilevel analyses reported above. Across both smaller trade-offs (e.g., missing work for a sick family member) and larger trade-offs (e.g., moving one’s family for a job), gendered patterns were generally consistent and aligned with the multilevel analyses. Men were more willing to prioritize work than women in two of the three work-prioritizing situations, whereas women were (marginally) more willing to prioritize family than men in all three family-prioritizing situations.

**Table 4:** Sacrificing Behaviors That Emerged From the Pilot Study

	*N*	% Women (of all participants mentioning this)
‘Sacrifice for work’ behaviors		
Occasionally working outside regular working hours (e.g., long hours, evenings, weekends)	67	43
Accepting additional responsibilities at work	27	44
*Moving your family for your job (to another city, state, or country)	13	31
‘Sacrifice for family’ behaviors		
Reducing work hours for your family	26	81
*Choosing not to seek out career enhancing opportunities (e.g., promotion, extra training)	20	35
Missing work to take care of a sick family member	17	29

Consistent with our hypotheses, women perceived higher family costs than did men in two of the three work-prioritizing situations. In one work-prioritizing situation (occasionally working outside regular hours), men perceived higher family benefits than women, aligning with H2d and representing the only deviation from the multilevel results. In contrast to our hypotheses, but consistent with the multilevel analyses, women also perceived higher career benefits than men in two work-prioritizing situations.

Regarding family-prioritizing situations, women perceived higher career costs than men and lower career benefits of prioritizing work in two of three situations, again aligning with multilevel analyses, indicating that women perceive prioritizing work as more detrimental for their career. Furthermore, as expected, women perceived higher family benefits than men in tow of three prioritizing family situations.

Notably, although not all individual prioritizing work situations and prioritizing family situations showed consistent significant gender differences in perceptions or willingness, no single situation disproportionately drove the overall patterns. Both larger sacrifices (e.g., moving one’s family for a job) and smaller, occasional sacrifices (e.g., occasionally working outside regular hours) reflected the general gendered tendencies. Taken together, these results suggest that gendered patterns of willingness to prioritize work versus family are evident across both smaller and larger trade-offs, although the magnitude and direction of perceived costs and benefits vary somewhat by specific situation.

## Discussion

Decades of research has shown that men tend to prioritize their breadwinning role, such as working full-time, taking fewer career breaks, and enduring longer commutes, while women tend to prioritize their caregiving role, by opting for part-time work, taking extended leave, and choosing more flexible jobs to accommodate family needs (Becker & Moen, 1999; Dahm et al., 2019; Eurostat, 2023; Herr et al., 2020). Our study tested whether gender differences in willingness to prioritize work or family indeed (still) exist and why they arise. We found that women were more willing than men to prioritize family and men more willing than women to prioritize work. Yet both reported a stronger willingness to prioritize family than to prioritize work.

To explain these patterns, we treated perceived costs and benefits as central constructs and tested two processes. First, consistent with the *gendered perceptions* explanation, women perceived greater family benefits than men when imagining prioritizing family and higher family costs when imagining prioritizing work. Women also perceived prioritizing family as more detrimental for their career and prioritizing work as more beneficial for their career than men. Overall these gendered perceptions partly explain why women were more willing to prioritize family and men to prioritize work.. Second, in line with the *gendered weighing explanation*, women’s willingness to prioritize family rose more sharply with higher perceived family benefits, whereas men’s willingness dropped more steeply with higher perceived career costs of prioritizing family. This suggests that women are especially sensitive to family benefits and men to career costs, although our cross-sectional design does not allow firm causal claims. Importantly, these findings were consistent across participants with and without children, suggesting that gendered decision processes are not limited to specific family contexts.

Our findings confirm and extend past work. First, our findings that both women and men are more willing to prioritize family than work, may reflect growing cultural appreciation for involved fatherhood and shifting norms around masculinity and caregiving. (Meeussen et al., 2019; Carlson et al., 2018; Galovan et al., 2014; Schieman et al., 2018).

Despite this general shift, we still find that men were still more willing than women to prioritize work and women more willing than men to prioritize family, consistent with broader literature on how gender stereotypes and norms lead men and women to make different work-family decisions. (Ellemers, 2018; Meeussen et al., 2021; Morgenroth et al., 2022; Ryan & Morgenroth, 2024). Our findings also align with earlier research on prioritizing family (sacrificing work for family) in which this was seen as costlier for men and more beneficial for women, and women themselves also indicate more willingness to prioritize family than men (Villanueva-Moya & Expósito, 2025) Previous research also showed that these greater benefits when prioritizing family are especially experienced by women high in communal strength (Villanueva-Moya & Expósito, 2025). Therefore, a communal motivation (which is consistent with the female stereotype) might make people more willing to make relational sacrifices and to enjoy them more (Kogan et al., 2010). Similarly, if caregiving is viewed and internalized as a feminine domain, men may see their involvement as less beneficial or even unnecessary. Future research should test to what extent internalized gender stereotypes indeed drive such gendered costs/benefits perceptions.

Unexpectedly, women perceived higher career costs than when prioritizing work over family. This contrasts with literature suggesting that men are penalized more when deviating from the provider role or ideal worker norms (Berdahl & Moon, 2013; Haines & Stroessner, 2019). One explanation is that women anticipate or experience stronger career penalties associated when revealing their parenthood identity at work, as documented in research on the motherhood penalty (Budig & England, 2001; Correll et al., 2007; Morgenroth & Heilman, 2017; Ridgeway & Correll, 2004; Yu & Hara, 2021). (Future research should disentangle whether these perceptions reflect actual gendered penalties or accumulated experiences (i.e., women having more experience with bringing sacrifices for their family).

Additionally, women in our study perceived higher career benefits when imagining prioritizing work over family than men, despite evidence that such sacrifices tend to yield fewer returns for women than for men (Ellemers, 2014; Heilman & Haynes, 2005). This contrasts with prior findings showing that women expect less success from career sacrifices (Meeussen et al., 2021). One explanation may lie in differences in context and measurement: whereas prior work focused on male-dominated fields and general willingness to sacrifice, our study examined concrete scenarios across a broader range of occupations. Such differences may activate different processes, with abstract judgments reflecting beliefs about structural inequality, and concrete scenarios eliciting more self-justifying reasoning, whereby individuals amplify expected returns to justify anticipated sacrifices (Kay & Friesen, 2011). Future research should further examine this mechanism.

At the same time, more career benefits perceived by women than men also means that men in our study perceived fewer career benefits from prioritizing work than women, This may reflect shifting standards (Biernat & Manis, 1994), whereby people evaluate others relative to different group-based expectations rather than against a common standard. Recent work shows that men are more likely than women to receive praise for prioritizing family responsibilities, precisely because such behavior is less expected of them (Meeussen et al., 2022). Therefore, men prioritizing work over family may also feel less visible or valued, precisely because such sacrifices are more expected from them than from women

Furthermore, our findings suggest that gendered weighing of family costs and family benefits may not play a role in small, everyday decisions, but become more influential when prioritizing family carries greater consequences (i.e. larger benefits or career costs). Under these circumstances, women were more willing than men to prioritize their family. Future research could test whether this pattern reflects internalized gender role expectations. For example, whether more traditional women feel more pressure to prioritize family than men and less traditional women when the perceived impact on their family is high. (Haines & Stroessner, 2019).

Taken together, our findings provide a mechanism-level based explanation of gendered work-family decision-making by examining cost-benefit perceptions and weighting processes. This approach bridges decision-making theories, work–family interface theories (e.g., Allen et al., 2000; Amstad et al., 2011; Van Steenbergen et al., 2007) and gender-role theories (Haines & Stroessner, 2019; Eagly & Karau, 2002). Rather than women and men having different costs/benefit perceptions of work-family trade-offs or women and men assigning different importance to family/career costs and benefits, we show that it is a combination of gendered perceptions and weighting. 

### Limitations and Future Research Directions

While our study provides novel insights into the psychological mechanisms behind gendered work-family prioritizations, several limitations warrant further investigation. Most notably, our study focused on individuals’ perceptions of costs and benefits and self-reported willingness to prioritize, rather than observing more objective costs and benefits or actual behaviors, reflecting “cold,” hypothetical reasoning rather than “hot,” real-time decision-making (based on the system 1, system 2 distinction; Kahneman, 2011), such perceptions may not always translate into real-life outcomes (Ajzen, 1991). However, the gendered pattern we observe in hypothetical scenarios, align with empirical evidence from naturalistic work–family decision-making contexts (e.g., Dahm et al., 2019). Nevertheless, testing causality and ecological validity remains important. Future research could examine whether manipulating people’s prioritization tendency (by inducing a work mode or family mode) indeed results in a different cost-benefit analysis and consequently, a different willingness to prioritize. Also, experimental work manipulating costs and benefits would allow a stronger causal test of the mechanisms we identified.Moreover, it remains unclear how accurate individuals estimate the costs and benefits of prioritizing work or family, or do they systematically over- or underestimate them? Future studies could examine whether individuals misperceive social expectations and associated costs or benefits (e.g., pluralistic ignorance; Miyajima & Yamaguchi, 2017; Munsch et al., 2014), and how this shapes decisions.

Future studies could also explore how gendered perceptions of costs and benefits emerge in the first place. To what extent are they shaped through internalized norms, vicarious learning (e.g., observing role models), personal interests, direct personal experiences, or some combination of these factors? Disentangling these influences would deepen our understanding of the origins of gendered decision-making.

Our model only partially explains the effect of gender on willingness to prioritize work or family, as a direct effect of gender remains even after accounting for perceived costs and benefits. This highlights the importance of considering additional factors, such as the relational context in which work-family decisions are made. Since such decisions often involve partners, future research should examine how partners influence each other’s willingness to sacrifice, through their own work-family priorities or responses to sacrifices (Zvonkovic et al., 1996). Gendered expectations about a partner’s reaction may be a key mechanism underlying persistent gender differences. For example, women with higher relationship commitment perceive greater benefits from prioritizing family (Villanueva-Moya & Expósito, 2025), consistent with research showing that women may adopt more traditional orientations when satisfied in heterosexual relationships (Sobol-Sarag et al., 2023).

A limitation of our approach is that, because each mediation model was fully saturated, we could not report fit indices. Thus, we could not determine whether a different model might provide a more optimal account of gendered willingness to prioritize, although our fully saturated, theory-driven models ensured that all hypothesized indirect effects were tested. Finally, although the gender differences we observed were small in effect size, they may accumulate over time to shape significant disparities in career progression, caregiving responsibilities, and broader gender inequality. Future longitudinal research is needed to examine whether these psychological mechanisms contribute to persistent gender gaps in work-family trajectories.

### Practice Implications

Understanding the psychological mechanisms behind gendered work-family decision-making provides actionable insights for policymakers, organizational leaders, and practitioners seeking to reduce gender inequality. First, interventions should explicitly counter the stereotype of mothers as “default caregivers” and fathers as “secondary helpers.” This would help both parents to see equal benefits of prioritizing family over work. Policy makers can implement equal and non-transferable parental leave to give fathers hands-on caregiving experience and confidence (Petts et al., 2020). This is likely to be most effective when combined with national media campaigns to normalize father involvement, as in Scandinavian countries where national media reinforced new norms (Klinth, 2008). Parenting organizations and community programs can also train and mentor fathers directly. For example, Program P by Promundo (Doyle et al., 2018) and the American National Fatherhood Initiative (Olshansky, 2006, as cited in Obure et al., 2020) demonstrating that programs training fathers’ caregiving skills and self-efficacy can lead to lasting changes in involvement and attitudes. Such changes can help rebalance perceptions and support more equal participation in both work and family life.

Second, counselors, HR professionals, and educators can help women and men to make decisions aligned with their own desires. For example, employers can normalize flexible work arrangements for women as well as men, thereby reducing stigma around men prioritizing caregiving. Career counselors and therapists can help individuals identify and challenge internalized gender norms, encouraging them to make decisions based on personal priorities rather than social norms and anticipated backlash. Furthermore, celebrating role models who break stereotypes (e.g., senior men taking parental leave or senior women prioritizing career progression) may enable individuals to make work-family choices unlimited by gender norms. Together, these implications underscore that interventions to promote gender equality cannot stop at offering “choices” to individuals but must also reshape the societal and organizational context in which those choices are made.

## Conclusion

Women and men live in an unequal reality where men are more rewarded, praised, and supported for prioritizing their work and women are more rewarded, praised and supported for prioritizing family, reinforcing traditional gender roles even as societal norms begin to shift. Our findings show that gender shapes the very process of work-family decision making, by impacting both the costs and benefits women and men anticipate and the importance they assign to these costs and benefits. Even when women and men face similar work/family choices, they do not make those choices on equal terms. Achieving meaningful progress toward gender equality therefore requires moving beyond expanding options for women and men to changing gendered expectations and standards that define which choices feel possible and valuable

## Supplementary Information

Supplementary Material 1.

## Data Availability

The data and R analysis script used in this study are publicly available at the Open Science Framework: https://osf.io/bkmzf/?view_only=0cede7d6a9d64baeba9f0233488b9b4e.
