# Supplementary material for: Prioritizing Work or Family? Investigating Women’s and Men’s Work-Family Decisions
Source: Sex Roles. 2026 Jul 8;92(4):31. doi: 10.1007/s11199-026-01663-0 (PMC13346125; doi:10.1007/s11199-026-01663-0)
Supplement: Supplementary file 1 — Supplementary Material 1. [file 11199_2026_1663_MOESM1_ESM.docx]

Online supplement for Aarntzen, L., Meeussen, L., Morgenroth, T., & Ryan, M. (2026). Prioritizing Work or Family? Investigating Women’s and Men’sWork-Family Decisions. Sex Roles. Lianne Aarntzen, Utrech University. Email: [e.m.j.aarntzen@uu.nl](mailto:e.m.j.aarntzen@uu.nl)

Pilot Study

Participants were recruited through Prolific, an online participant recruitment website which, in comparison to other online research platforms, generally provides good quality data in terms of scale reliability, replication of known effects, passing attention checks, participants’ (low) familiarity with measures, and honesty of participants (Peer et al., 2017). The aim was to collect responses from 80 participants who were asked to list behaviors that benefit their family or career. Seven participants were rejected because they provided irrelevant content and were replaced by Prolific with seven other participants. The final sample consisted of 80 employed participants (48.8% women, 51.2% men). All participants were fluent in English and most were in a relationship (86.2%). They were on average 36.56 years old (SD = 9.35), had 1.66 children (SD = 0.78), and the average age of their youngest child was 8.06 years old (SD = 6.33). Participants worked on average 36.47 hours per week (SD = 8.87).

Participants were asked to list three behaviors that benefited their career but may have had costs for their family, and three behaviors that benefited their family but may have been costly for their career. In total, 456 responses were received. These were categorized in 22 thematic clusters (e.g., the response ‘working while being on holiday’ was classified under the theme ‘work during family time’). After this clustering, we further narrowed down the list of behaviors and selected the behaviors that were mentioned frequently by both men and women, were applicable to all types of jobs, were applicable to different countries, covered a diversity of consequences from minor to major consequences, and were distributed equally in behaviors that represented a sacrifice for work or for family. This resulted in a total of six behaviors listed in Table S1.

Table S1

Sacrificing Behaviors that Emerged From the Pilot Study

|  | N | % Women (of all participants mentioning this) |
| --- | --- | --- |
| ‘Sacrifice for work’ behaviors |  |  |
| Occasionally working outside regular working hours (e.g., long hours, evenings, weekends) | 67 | 43 |
| Accepting additional responsibilities at work | 27 | 44 |
| *Moving your family for your job (to another city, state, or country) | 13 | 31 |
| ‘Sacrifice for family’ behaviors |  |  |
| Reducing work hours for your family | 26 | 81 |
| *Choosing not to seek out career enhancing opportunities (e.g., promotion, extra training) | 20 | 35 |
| Missing work to take care of a sick family member | 17 | 29 |

Table S2

Results of Multilevel Modeling on Willingness to Sacrifice

| Level and Variable | Null  (Step 1) | Random Intercept and Fixed Slope (Step 2) | Cross-Level Interaction (Step 3) |
| --- | --- | --- | --- |
| Level 1 |  |  |  |
| Intercept | 4.51** (0.029) | 4.16**(0.10) | 4.35**(0.06) |
| Sacrifice domain |  | 0.57** (0.06) | 0.27**(0.08) |
| Level 2 |  |  |  |
| Gender |  | 0.04 (0.06) | -0.26**(0.08) |
| Cross-level interaction |  |  |  |
| Gender * Sacrifice Domain |  |  | 0.60**(0.12) |
| Variance components |  |  |  |
| Within-person variance | 3.36 (1.83) | 3.28 (1.81) | 3.25 (1.80) |
| Intercept variance | 0.00 (0.00) | 0.00 (0.00) | 0.00 (0.00) |
| -2 log likelihood (FIML) | 15184.9 | 15092.9 | 15033.4 |
| Number of estimated parameters | 2 | 4 | 7 |
| Pseudo R^2^ | 0.02 | 0.02 | 0.03 |

Note. FIML = full information maximum likelihood estimation; L1 = Level 1; L2 = Level 2. L1 N = 3750 and L2 sample size = 625. Values in parentheses are standard errors.

*p < .05. **p < .01.

Table S3.

Results of Multilevel Modeling on Benefit Career

| Level and Variable | Null  (Step 1) | Random Intercept and Fixed Slope (Step 2) | Cross-Level Interaction (Step 3) |
| --- | --- | --- | --- |
| Level 1 |  |  |  |
| Intercept | 3.99** (0.04) | 5.68** (0.04) | 5.54** (0.05) |
| Domain |  | -3.33**(0.05) | -3.05** (0.07) |
| Level 2 |  |  |  |
| Gender |  | -0.05 (0.05) | 0.23** (0.07) |
| Cross-level interaction |  |  |  |
| Gender * Domain |  |  | -0.56** (0.09) |
| Variance components |  |  |  |
| Within-person variance | 4.91 (2.22) | 2.03 (1.43) | 2.01 (1.42) |
| Intercept variance | 0.00 (0.00) | 0.10 (0.31) | 0.10 (0.32) |
| -2 log likelihood (FIML) | 16606.3 | 13462.6 | 13425.8 |
| Number of estimated parameters | 3 | 5 | 6 |
| Pseudo R^2^ | 0.00 | 0.57 | 0.57 |

Note. FIML = full information maximum likelihood estimation; L1 = Level 1; L2 = Level 2. L1 N = 3750 and L2 sample size = 625. Values in parentheses are standard errors.

*p < .05. **p < .01.

Table S4.

Results of Multilevel Modeling On Cost Career

| Level and Variable | Null  (Step 1) | Random Intercept and Fixed Slope (Step 2) | Cross-Level Interaction (Step 3) |
| --- | --- | --- | --- |
| Level 1 |  |  |  |
| Intercept | 3.71** (0.03) | 2.39** (0.53) | 2.50** (0.06) |
| domain |  | 2.55** (0.05) | 2.33** (0.07) |
| Level 2 |  |  |  |
| Gender |  | 0.09 (0.07) | -0.14 (0.10) |
| Cross-level interaction |  |  |  |
| Gender * Domain |  |  | 0.45** (0.10) |
| Variance components |  |  |  |
| Within-person variance | 4.39 (2.10) | 2.49 (1.58) | 2.47 (1.57) |
| Intercept variance | 0.00 (0.00) | 0.27 (0.52) | 0.27 (0.53) |
| -2 log likelihood (FIML) | 16189.4 | 14377.1 | 14357.7 |
| Number of estimated parameters | 3 | 5 | 6 |
| Pseudo R^2^ | 0.00 | 0.37 | 0.37 |

Note. FIML = full information maximum likelihood estimation; L1 = Level 1; L2 = Level 2. L1 N = 3750 and L2 sample size = 625. Values in parentheses are standard errors.

*p < .05. **p < .01.

Table 5.

Results of Multilevel Modeling On Benefit Family

|  |  |  |  |
| --- | --- | --- | --- |
| Level and Variable | Null  (Step 1) | Random Intercept and Fixed Slope (Step 2) | Cross-Level Interaction (Step 3) |
| Level 1 |  |  |  |
| Intercept | 4.25** (0.03) | 3.32** (0.05) | 3.45** (0.06) |
| domain |  | 1.75** (0.06) | 1.48** (0.08) |
| Level 2 |  |  |  |
| Gender |  | 0.11 (0.06) | -0.16 (0.08) |
| Cross-level interaction |  |  |  |
| Gender * Domain |  |  | 0.54** (0.12) |
| Variance components |  |  |  |
| Within-person variance | 4.12 (2.03) | 3.36 (1.83) | 3.37 (1.83) |
| Intercept variance | 0.00 (0.00) | 0.00 (0.00) | 0.00 (0.00) |
| -2 log likelihood (FIML) | 15952.8 | 15181.6 | 15083.1 |
| Number of estimated parameters | 3 | 5 | 6 |
| Pseudo R^2^ | 0.00 | 0.19 | 0.19 |

Note. FIML = full information maximum likelihood estimation; L1 = Level 1; L2 = Level 2. L1 N = 3750 and L2 sample size = 625. Values in parentheses are standard errors.

*p < .05. **p < .01.

Table 6.

Results of Multilevel Modeling On Cost Family

|  |  |  |  |
| --- | --- | --- | --- |
| Level and Variable | Null  (Step 1) | Random Intercept and Fixed Slope (Step 2) | Cross-Level Interaction (Step 3) |
| Level 1 |  |  |  |
| Intercept | 4.13** (0.03) | 4.84** (0.05) | 4.75** (0.06) |
| Domain |  | -1.56** (0.07) | -1.36** (0.08) |
| Level 2 |  |  |  |
| Gender |  | 0.13 (0.07) | 0.33** (0.09) |
| Cross-level interaction |  |  |  |
| Gender * Domain |  |  | -0.40** (0.11) |
| Variance components |  |  |  |
| Within-person variance | 3.77 (1.94) | 3.04 (1.74) | 3.03 (1.74) |
| Intercept variance | 0.06 (0.25) | 0.18 (0.42) | 0.18 (0.42) |
| Pseudo R^2^ | 0.00 | 0.16 | 0.16 |

Note. FIML = full information maximum likelihood estimation; L1 = Level 1; L2 = Level 2. L1 N = 3750 and L2 sample size = 625. Values in parentheses are standard errors.

*p < .05. **p < .01.
